# Correction: Application of the skills network approach to measure physician competence in shared decision making based on self-assessment

**DOI:** 10.1371/journal.pone.0294211

**Published:** 2023-11-03

**Authors:** Levente Kriston, Lea Schumacher, Pola Hahlweg, Martin Härter, Isabelle Scholl

There are errors in the Funding section. The correct Funding statement is: The original study, of which data were used for analysis, was funded by the German Federal Ministry of Education and Research (https://www.bmbf.de/bmbf/en/home/home_node.html, grant number: 01GX0742, grant received by LK, MH and IS). The secondary analysis presented here was not externally funded. The funder of the original study had no role in study design, data collection and analysis, decision to publish, or preparation of the manuscript. We acknowledge financial support for the publication costs from the Open Access Publication Fund of the University Medical Center Hamburg-Eppendorf (UKE) and the German Research Foundation (DFG).
