# Supplementary material for: Application of the skills network approach to measure physician competence in shared decision making based on self-assessment
Source: PLoS One. 2023 Feb 27;18(2):e0282283. doi: 10.1371/journal.pone.0282283 (PMC9970074; doi:10.1371/journal.pone.0282283)
Supplement: S1 File — (PDF) [file pone.0282283.s001.pdf]

Supplement to "Application of the skills network approach to measure physician competence in shared decision making based on self-assessment" by Kriston L et al.

S1 File. Information on Prior Distributions.

MULTILEVEL ANALYSES OF SDM-Q-DOC DATA

We chose weakly informative prior distribution which granted the most probability to plausible values.

Population intercept: As all predictors were centred, the intercept was expected to be positive. Further, previous studies and a similar analysis of patient-reported data (Kriston et al., 2020) showed that items of the SDM-Q-9 and SDM-Doc are often scored rather high. Therefore, a normal distribution with a mean of 4 and a standard deviation of 2 was chosen. This grants most probability to values between 3 and 5.

Normal (4,2)

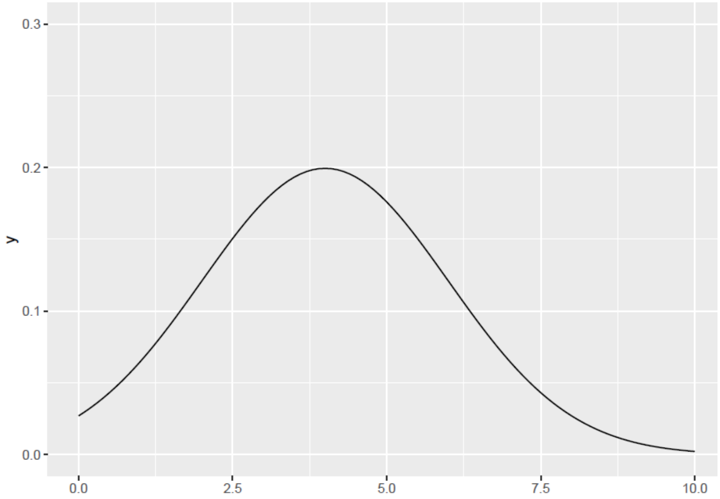

Population slope: Previous analyses with patient data showed that slopes ranged mostly between -1 and 1 (and strong multicollinearity can be expected between items). Therefore, a normal distribution with a mean of 0 and a standard deviation with 2 was chosen.

Normal (0,2)

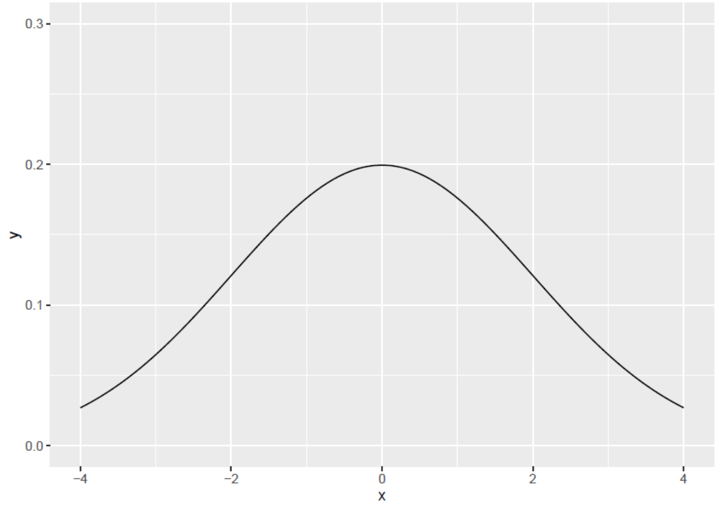

Standard deviation of the group level effects and standard deviation of the population effect: As these values were rather small in the analysis of the patient data, a halfCauchy distribution was chosen, granting most probability to small positive values while also allowing for large positive values.

HalfCauchy(0,4)

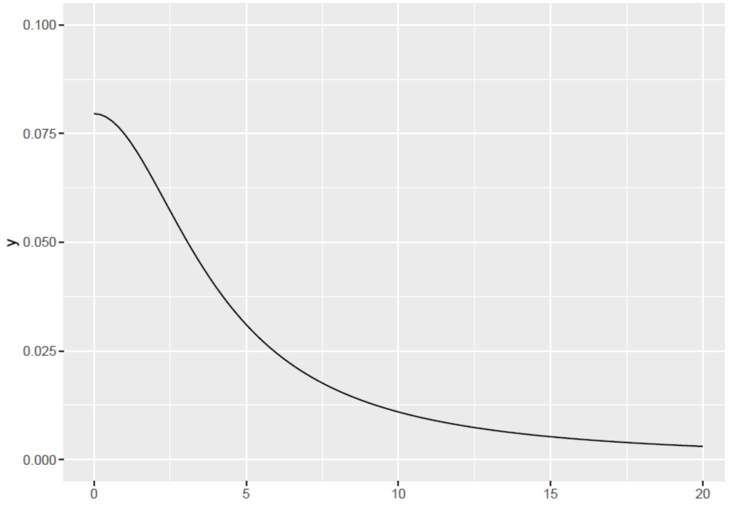

PREDICTION OF OBSERVER-RATED MEASURES FROM NETWORK PARAMETERS

Confirmatory analyses

The prior distributions were set for each parameter based on means and standard deviations that were observed in the analysis with the patient-reported data (Kriston et al., 2020).

OPTION 12

Intercept: normal(16.15,0.86)

Activation Skill 1: normal(35.66, 43.53)
Activation skill 6: normal(62.74 ,26.15)
Activation skill 7: normal(1.02, 22.33)

Outstrength skill 1: normal(-7.11 ,5.20)
Outstrength skill 6: normal(9.48, 5.38)
Outstrength skill 7: normal(-11.21, 4.05)

Instrength skill 1: normal(13.75, 30.63)
Instrength skill 6: normal(5.49, 5.20)
Instrength skill 7: normal(-1.5, 5.32)

OPTION 5

Intercept: normal(12.44,1.24),

Activation Skill 1: normal(-31.30,62.69)
Activation skill 6: normal(54.99,37.64),
Activation skill 7: normal(14.67,32.16),

Outstrength skill 1: normal(-6.02,7.49)
Outstrength skill 6: normal(-0.17,7.76),
Outstrength skill 7: normal(-10.46,5.83)

Instrength skill 1: normal(17.21,44.12)
Instrength skill 6: normal(15.05,7.49)
Instrength skill 7: normal(-0.61,7.67)

HCS4

Intercept: normal(33.20,0.59),

Activation Skill 1: normal(-34.35,29.85),
Activation skill 6: normal(36.71,17.92),
Activation skill 7: normal(27.77,15.31),

Outstrength skill 1: normal(-4.55,3.57),
Outstrength skill 6: normal(11.75,3.69),
Outstrength skill 7: normal(-3.62,2.77)

Instrength skill 1: normal(13.25,20.98)
Instrength skill 6: normal(3.81,3.56)
Instrength skill 7: normal(-4.27,3.65)

Exploratory analyses

Weakly informative priors were chosen which granted most probability to plausible values.

Intercept: As all predictors are centred, we expect a positive intercept between 0 and 100. Further, the analysis with the patient-reported data showed intercepts around 20, therefore, we chose a normal distribution with a mean of 20 and a standard deviation of 40. This grants relatively high probability to values between 0 and 50.

Normal (20,40)

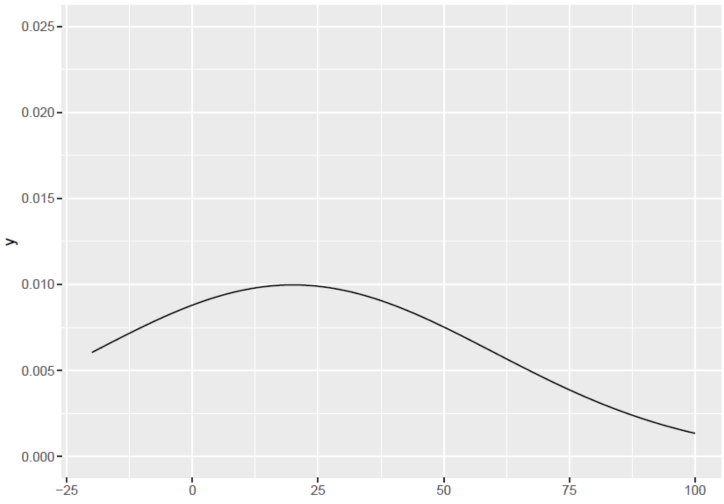

Slopes: Due to the scale (0-100) and the analysis with the patient-reported data, we expected slopes to fall between -100 and 100 with slightly higher probability of being closer to 0. Therefore, we chose a normal distribution with a mean of 0 and a standard deviation of 100 granting roughly equal probability to values between -100 and 100

Normal (0,100)

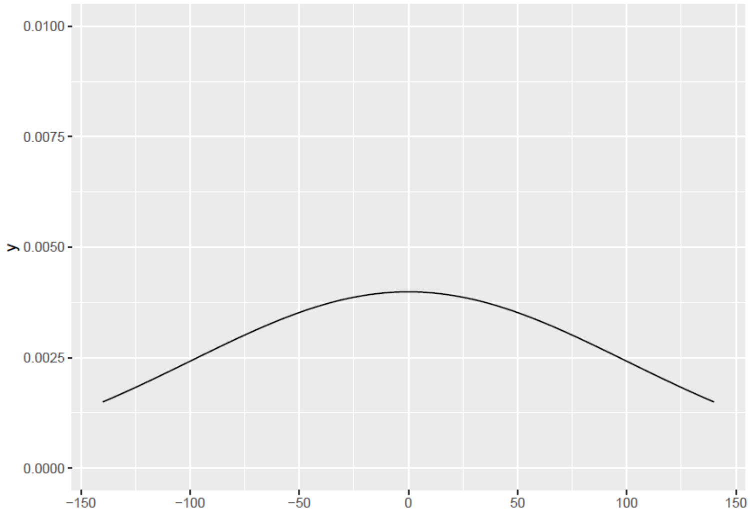

REFERENCES

Kriston L, Hahlweg P, Härter M, Scholl I. A skills network approach to physicians' competence in shared decision making. Health Expect. 2020;23(6):1466-1476. doi:10.1111/hex.13130
