# Supplementary material for: Application of the skills network approach to measure physician competence in shared decision making based on self-assessment
Source: PLoS One. 2023 Feb 27;18(2):e0282283. doi: 10.1371/journal.pone.0282283 (PMC9970074; doi:10.1371/journal.pone.0282283)
Supplement: S1 Fig — (PDF) [file pone.0282283.s002.pdf]

S1 Fig. Skills Networks of Individual Physicians.

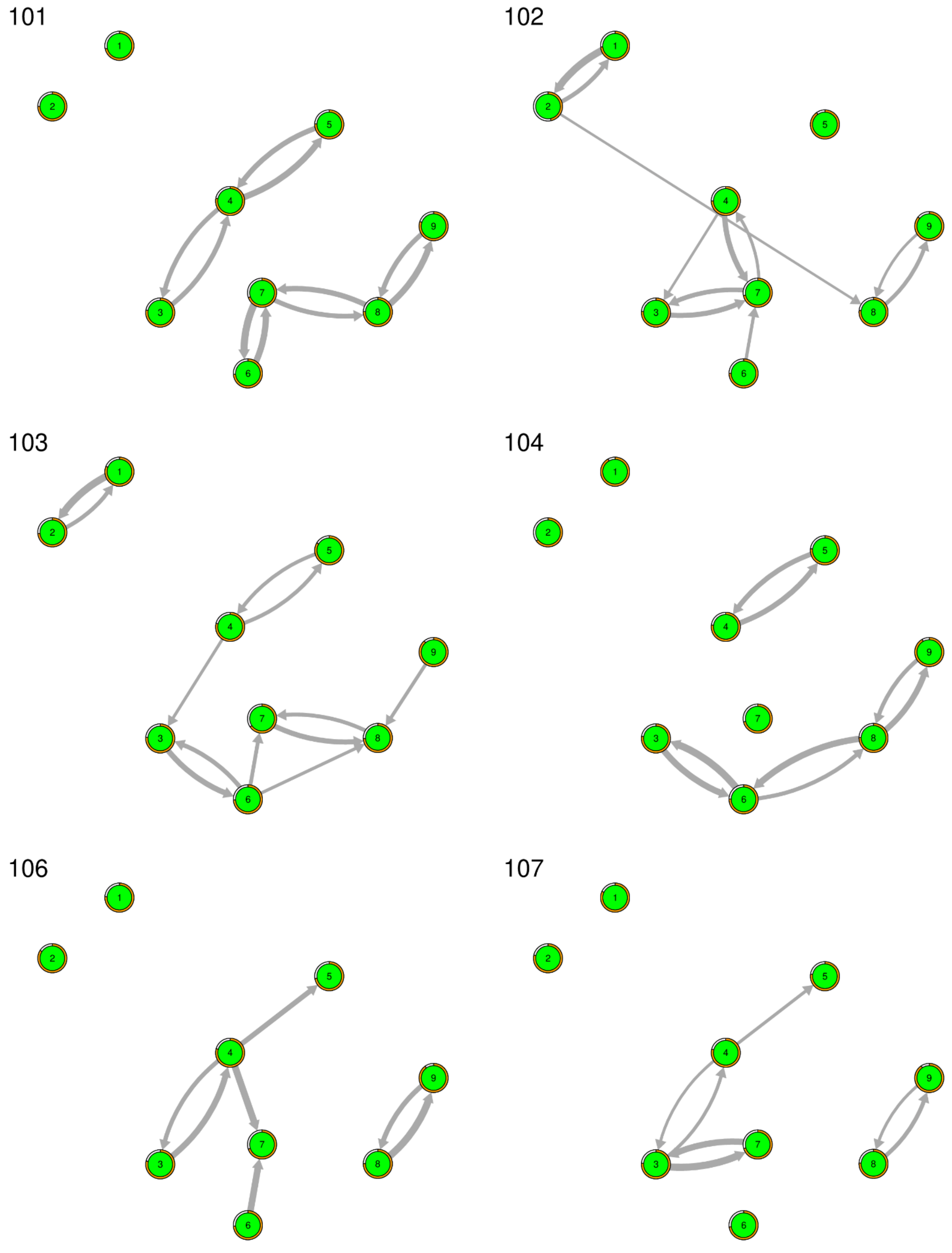

108

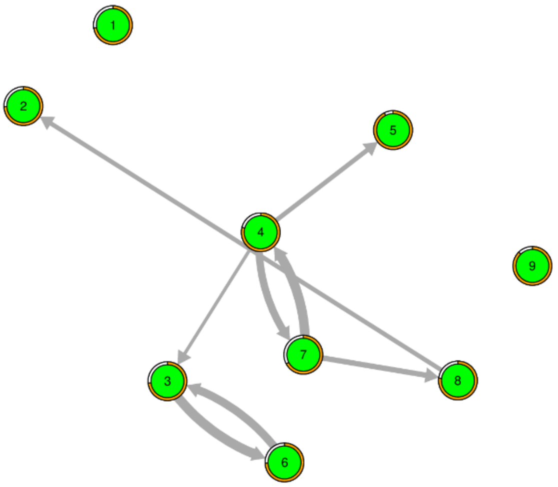

109

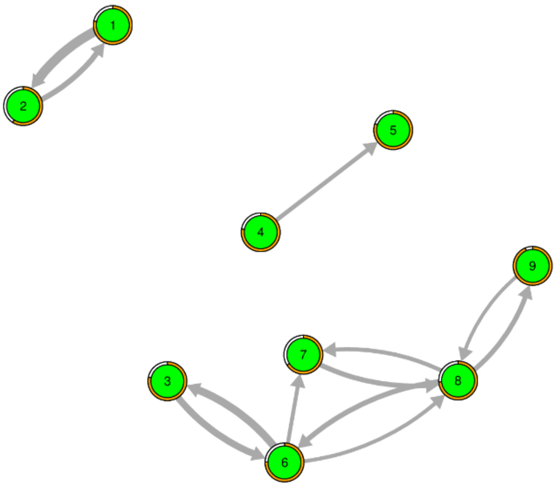

110

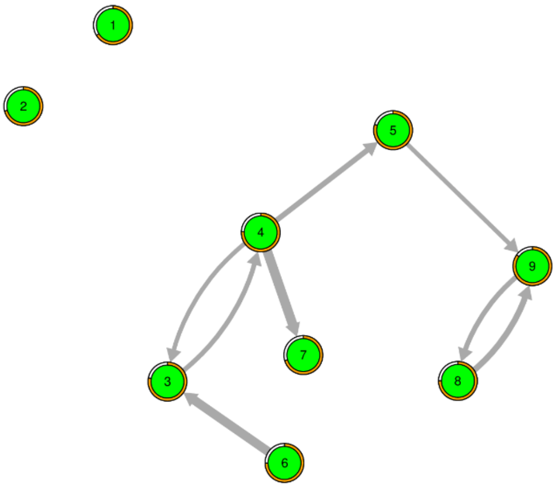

112

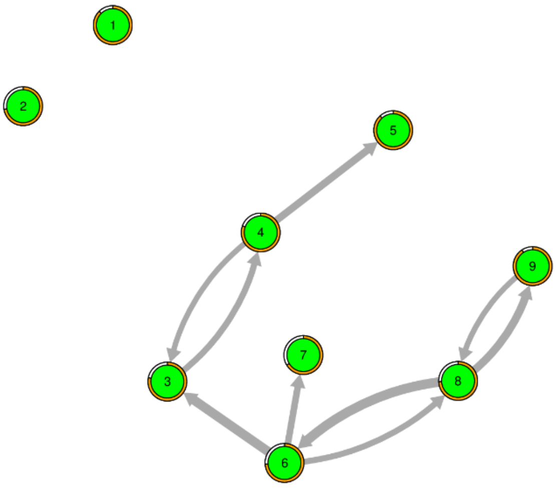

113

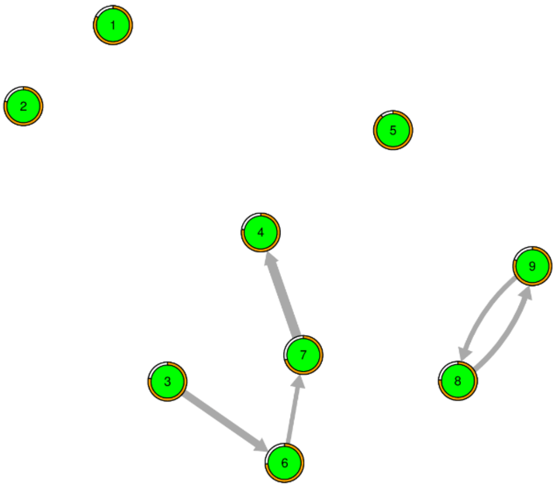

115

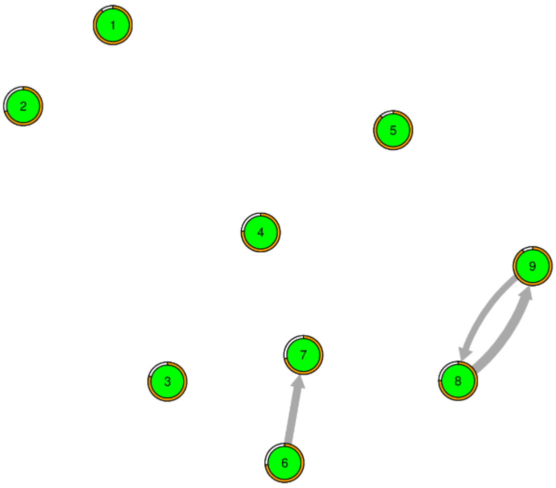

116

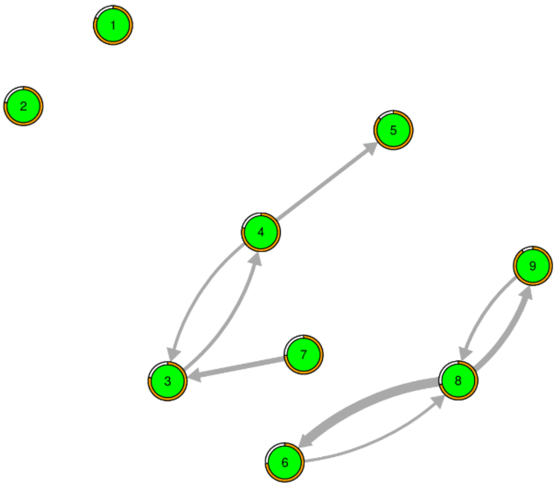

117

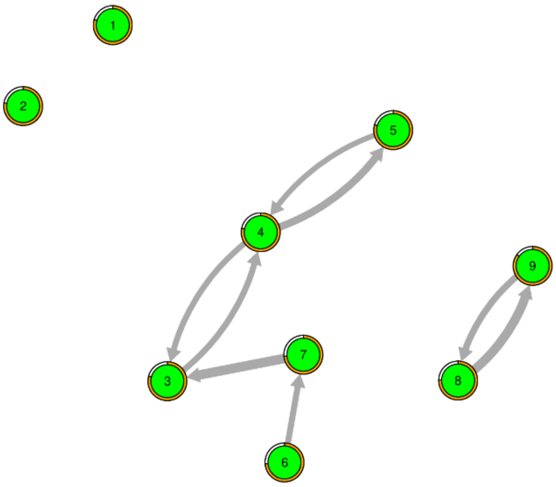

201

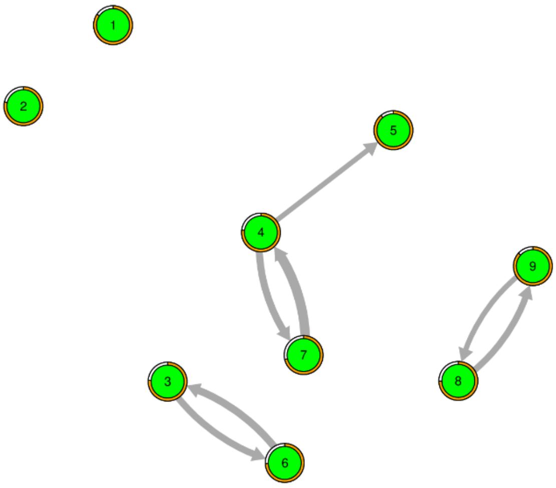

203

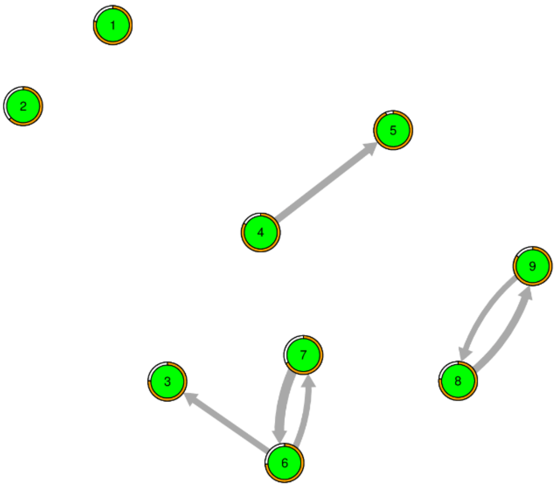

204

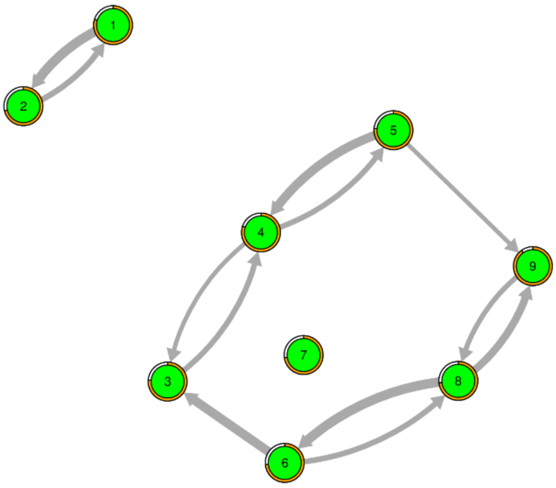

205

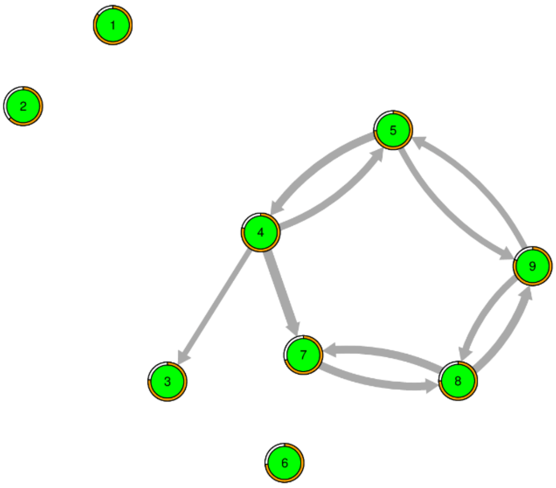

206

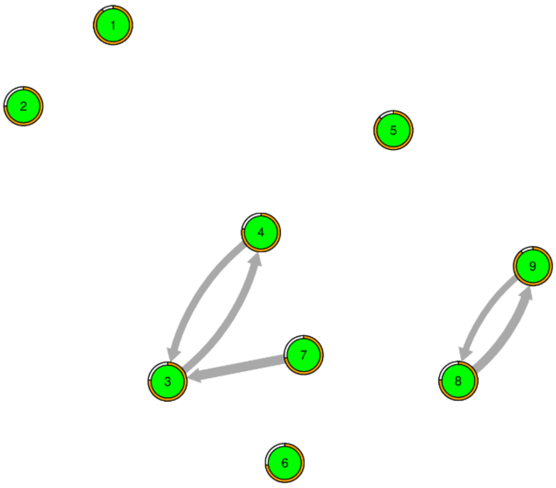

301

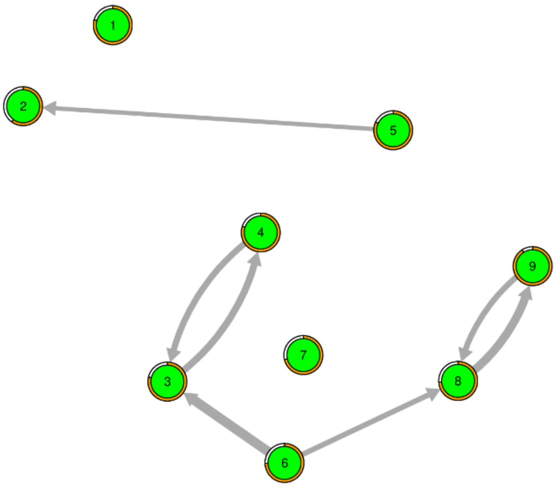

302

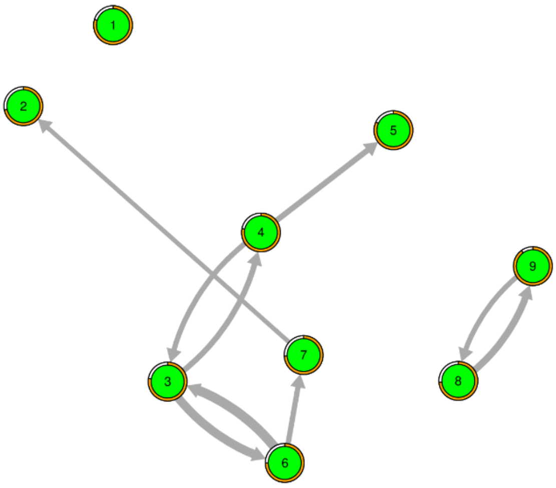

303

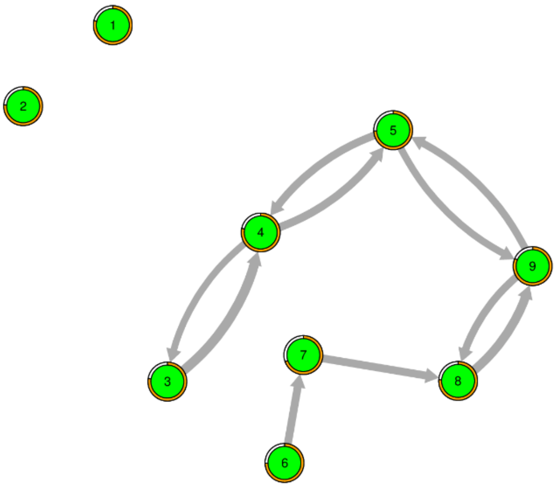

304

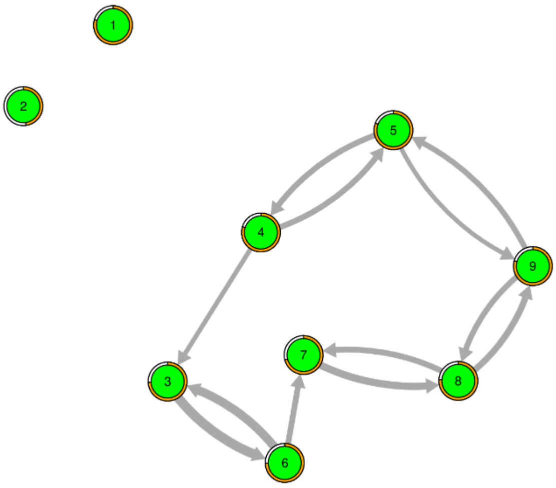

401

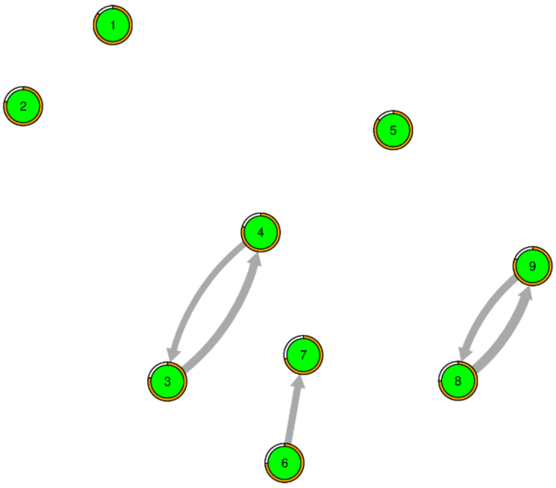

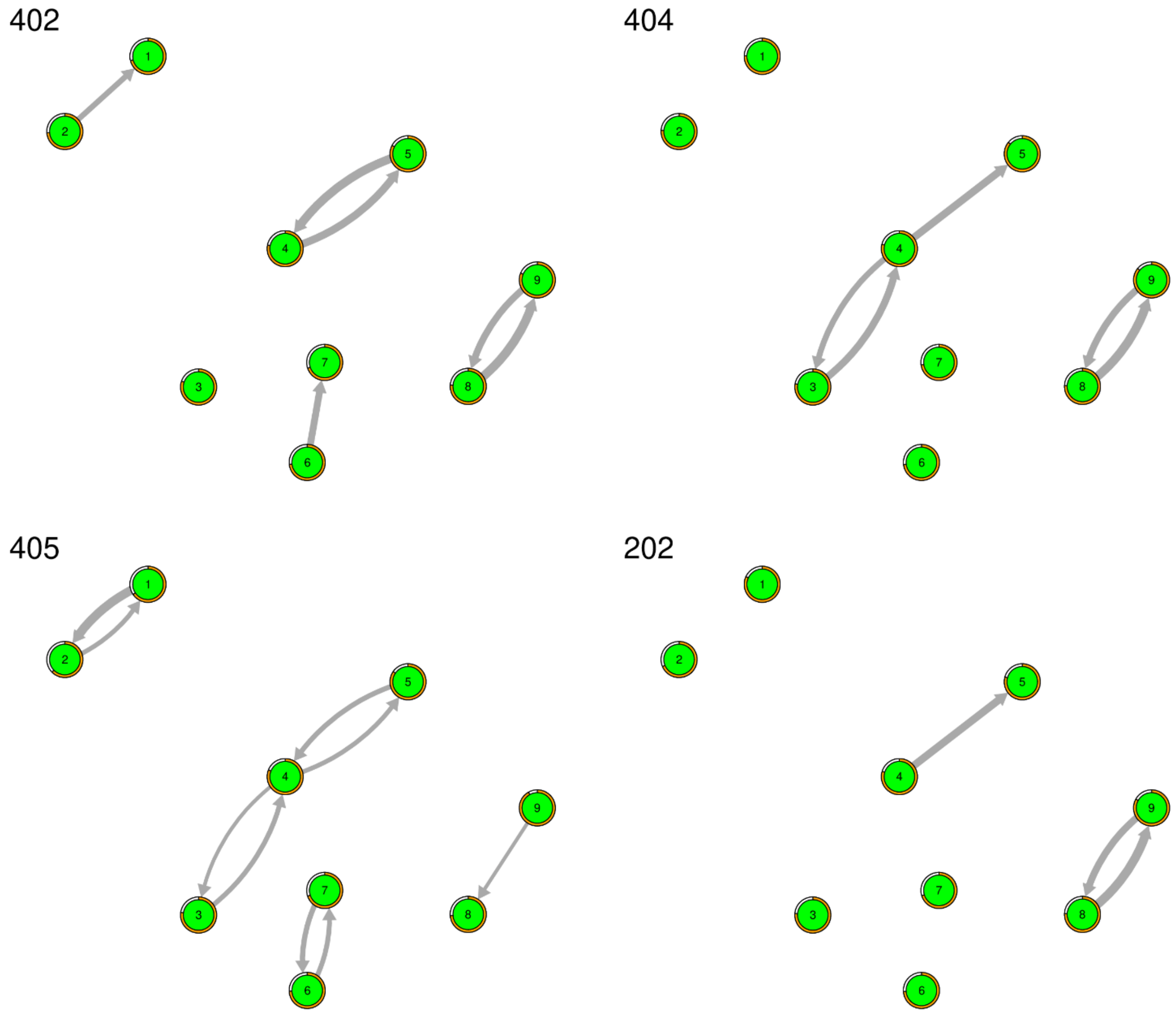

Width of the arrows are relative to the strength of the association between the respective skills. The pie around each node indicates the activation of each item. Skill 1 = focusing the decision, Skill 2 = sharing the decision, Skill 3 = presenting options, Skill 4 = informing on options, Skill 5 = supporting comprehension, Skill 6 = eliciting preferences, Skill 7 = deliberating the decision, Skill 8 = selecting an option, Skill 9 = planning actions. The position of the nodes is fixed (aligned according to the population network) for better comparability.
