# Supplementary material for: Application of the skills network approach to measure physician competence in shared decision making based on self-assessment
Source: PLoS One. 2023 Feb 27;18(2):e0282283. doi: 10.1371/journal.pone.0282283 (PMC9970074; doi:10.1371/journal.pone.0282283)
Supplement: S1 Table — (PDF) [file pone.0282283.s003.pdf]

Supplement to "Application of the skills network approach to measure physician competence in shared decision making based on self-assessment" by Kriston L et al.

S1 Table. Prediction of Observer-Rated Shared Decision Making Competence from the Activation of All Skills.

**Activation**	**Option 12 (n=22)**	**Option 5 (n=24)**	**4HCS (n=22)**
	**Estimate [95% CI]**	**Estimate [95% CI]**	**Estimate [95% CI]**
Intercept	16.81 [14.88 to 18.96]	12.38 [10.46 to 14.30]	33.38 [31.57 to 35.16]
Skill 1	7.55 [1.90 to 13.13]*	8.35 [2.24 to 13.95]*	2.33 [-2.93 to 7.72]
Skill 2	5.23 [-0.35 to 10.52]	3.00 [-2.48 to 8.07]	2.75 [-2.05 to 7.46]
Skill 3	3.30 [-25.65 to 31.17]	28.80 [0.47 to 55.75]*	-4.59 [-31.24 to 21.95]
Skill 4	22.32 [-5.81 to 48.70]	35.69 [8.89 to 61.25]*	7.43 [-17.10 to 31.30]
Skill 5	-4.88 [-12.65 to 2.70]	2.31 [-4.64 to 9.20]	-2.44 [-9.44 to 4.05]
Skill 6	98.99 [15.33 to 173.56]*	156.74 [67.02 to 228.91]*	39.01 [-31.95 to 107.67]
Skill 7	2.54 [-15.04 to 19.33]	8.78 [-9.94 to 25.73]	0.87 [-15.47 to 16.38]
Skill 8	3.05 [-43.56 to 44.73]	3.20 [-39.08 to 41.74]	11.25 [-28.83 to 49.44]
Skill 9	-2.20 [-12.98 to 8.97]	-6.71 [-17.70 to 4.79]	6.10 [-3.94 to 15.99]
R^2	0.564	0.608	0.419
R	0.751	0.780	0.647

Note. Skill 1 = focusing the decision, Skill 2 = sharing the decision, Skill 3 = presenting options, Skill 4 = informing on options, Skill 5 = supporting comprehension, Skill 6 = eliciting preferences, Skill 7 = deliberating the decision, Skill 8 = selecting an option, Skill 9 = planning actions.
* With a probability of at least 95%, this parameter is different from zero.
