# Supplementary material for: Application of the skills network approach to measure physician competence in shared decision making based on self-assessment
Source: PLoS One. 2023 Feb 27;18(2):e0282283. doi: 10.1371/journal.pone.0282283 (PMC9970074; doi:10.1371/journal.pone.0282283)
Supplement: S2 Table — (PDF) [file pone.0282283.s004.pdf]

Supplement to "Application of the skills network approach to measure physician competence in shared decision making based on self-assessment" by Kriston L et al.

S2 Table. Prediction of Observer-Rated Shared Decision Making Competence from the Outstrength of All Skills.

**Outstrength**			
	**Option 12 (n=22)**	**Option 5 (n=24)**	**4 HCS (n=22)**
	**Estimate [95% CI]**	**Estimate [95% CI]**	**Estimate [95% CI]**
Intercept	15.81 [13.57 to 18.16]	11.70 [9.44 to 13.99]	32.70 [30.92 to 34.45]
Skill 1	-16.87 [-53.41 to 17.16]	-11.74 [-49.50 to 29.63]	-10.92 [-37.13 to 16.69]
Skill 2	3.22 [-19.67 to 26.09]	1.81 [-23.32 to 26.03]	2.38 [-14.99 to 19.93]
Skill 3	-0.91 [-8.56 to 7.25]	0.75 [-8.61 to 10.21]	-1.18 [-7.42 to 5.53]
Skill 4	-5.46 [-16.40 to 5.88]	-10.94 [-21.50 to -0.10]*	-8.54 [-17.43 to 0.35]
Skill 5	-2.25 [-16.15 to 12.87]	2.34 [-13.82 to 19.12]	1.69 [-9.55 to 12.91]
Skill 6	4.79 [-3.44 to 12.53]	7.63 [-1.24 to 16.62]	2.94 [-3.15 to 9.25]
Skill 7	-3.25 [-11.51 to 4.73]	-3.69 [-12.06 to 3.92]	0.31 [-6.15 to 6.53]
Skill 8	7.91 [-5.65 to 21.76]	-0.44 [-15.82 to 14.92]	-2.80 [-13.41 to 7.65]
Skill 9	-3.52 [-22.39 to 14.74]	-1.25 [-17.38 to 14.79]	-12.27 [-26.24 to 2.22]
R^2	0.542	0.543	0.467
R	0.736	0.737	0.683

Note. Skill 1 = focusing the decision, Skill 2 = sharing the decision, Skill 3 = presenting options, Skill 4 = informing on options, Skill 5 = supporting comprehension, Skill 6 = eliciting preferences, Skill 7 = deliberating the decision, Skill 8 = selecting an option, Skill 9 = planning actions.

* With a probability of at least 95%, this parameter is different from zero
