# Supplementary material for: Application of the skills network approach to measure physician competence in shared decision making based on self-assessment
Source: PLoS One. 2023 Feb 27;18(2):e0282283. doi: 10.1371/journal.pone.0282283 (PMC9970074; doi:10.1371/journal.pone.0282283)
Supplement: S3 Table — (PDF) [file pone.0282283.s005.pdf]

Supplement to "Application of the skills network approach to measure physician competence in shared decision making based on self-assessment" by Kriston L et al.

S3 Table. Prediction of Observer-Rated Shared Decision Making Competence from the Instrength of All Skills.

Instrength

	Option 12 (n=22)	Option 5 (n=24)	4HCS (n=22)
	**Estimate [95% CI]**	**Estimate [95% CI]**	**Estimate [95% CI]**
Intercept	15.82 [13.85 to 17.84]	11.54 [8.86 to 13.99]	33.15 [31.49 to 34.87]
Skill 1	5.68 [-10.09 to 21.91]	-3.41 [-26.49 to 20.05]	11.02 [-3.01 to 25.65]
Skill 2	-18.10 [-43.27 to 5.67]	-6.58 [-42.02 to 28.19]	-19.66 [-41.28 to 0.87]
Skill 3	5.05 [-3.07 to 13.04]	-1.69 [-12.38 to 9.91]	2.24 [-4.66 to 8.80]
Skill 4	-8.13 [-15.24 to -1.24]*	-3.73 [-12.23 to 4.93]	-3.49 [-9.39 to 2.47]
Skill 5	5.90 [-7.05 to 19.53]	-5.57 [-18.99 to 8.01]	1.15 [-9.87 to 12.12]
Skill 6	5.15 [-1.33 to 11.58]	9.19 [0.73 to 17.97]*	2.22 [-3.61 to 7.56]
Skill 7	1.97 [-5.34 to 9.84]	2.09 [-7.26 to 11.41]	-0.44 [-6.87 to 6.09]
Skill 8	3.49 [-2.22 to 9.28]	3.91 [-3.75 to 11.96]	-1.26 [-6.05 to 3.44]
Skill 9	-1.81 [-25.44 to 20.98]	8.82 [-19.38 to 35.46]	-3.84 [-23.80 to 15.31]
R^2	0.594	0.496	0.479
R	0.771	0.704	0.692

Note. Skill 1 = focusing the decision, Skill 2 = sharing the decision, Skill 3 = presenting options, Skill 4 = informing on options, Skill 5 = supporting comprehension, Skill 6 = eliciting preferences, Skill 7 = deliberating the decision, Skill 8 = selecting an option, Skill 9 = planning actions.

* With a probability of at least 95%, this parameter is different from zero
