# Supplementary material for: Application of the skills network approach to measure physician competence in shared decision making based on self-assessment
Source: PLoS One. 2023 Feb 27;18(2):e0282283. doi: 10.1371/journal.pone.0282283 (PMC9970074; doi:10.1371/journal.pone.0282283)
Supplement: Attachment — Submitted filename: Response to Reviewers.pdf [file pone.0282283.s006.pdf]

Response to Reviewers

Reviewer 1

1. “The article focuses on the assessment whether using the skills network approach allows for predicting observer-rated shared decision making (SDM) competence of physicians from their self-reported SDM skills. The article is well written, methodologically well done, and focuses on an important topic. However, I would like to suggest some points that should be added or slightly changed.”

- Thank you very much for the overall positive assessment of our study.

2. “Abstract - Conclusion: You conclude that SDM skill ratings offer new theoretically and empirically grounded opportunities for the assessment of SDM competence in routine medical care. Please add practical implications.”

- Thank you for this comment. We added information on the practical implications of the skills network approach for SDM in the Abstract, p.3.

3. “Introduction: The introduction is written very well. I only suggest to add some sentences with regard to the worth of shared decision making (SDM) in medical consultations.”

- We added a paragraph at the beginning of the Introduction on the value of SDM describing its ethical imperative and possible benefits, p.4.

4. “Methods: I suggest to display your study design and measures graphically. This would give the reader a good overview on the design and the different measures.”

- Thank you very much for this idea. We created a figure on the design, measures and analysis which we added in the Methods section, p.6 Figure 1.

5. “Results: The presentation of results is well done and well implemented in tabular and graphical form. No further suggestions.”

- Thank you for this positive feedback.

6. “Discussion: From my point of view the discussion section should be widened. I suggest to add some practical implications that can be drawn from your results.”

- We extended the Discussion section in regards to theoretical and practical implications of the skills network approach to SDM, p.15.

7. “Please discuss collinearity between network parameters in more detail.

- Within the Discussion section, we further specified the limitations due to collinearity and the need for more research on individual network parameters, p.16.

8. “Conclusion: From my point of view, I suggest leaving out the references in the summary. They should rather appear in the discussion and support it.”

- Thank you for this comment. We incorporated the references within the Discussion section and rewrote the conclusion without references, p. 17.

9. "Data Availability: Please give some more explanations for the restrictions that apply."

- The restrictions are necessary due to the limited resources that are (and expected to be) available for the workload associated with data sharing.

10. "I am wondering why your analyses are based on data collected in 2009/2010. Could you shortly explain the time delay."

- As this study is a secondary data analysis, the original data collection (and the main analysis) of the data is quite long ago. Additionally, the skills network approach to SDM was only recently developed (Kriston L, Hahlweg P, Härter M, Scholl I. A skills network approach to physicians' competence in shared decision making. Health Expect. 2020;23: 1466–1476. doi:10.1111/hex.13130). Therefore, we were interested in reanalyzing this data using this new approach.
