# Supplementary material for: Application of the skills network approach to measure physician competence in shared decision making based on self-assessment
Source: PLoS One. 2023 Feb 27;18(2):e0282283. doi: 10.1371/journal.pone.0282283 (PMC9970074; doi:10.1371/journal.pone.0282283)
Supplement: Attachment — Submitted filename: Response to reviewers.pdf [file pone.0282283.s007.pdf]

Response to reviewers

#1 Based on their previously published method, they aimed to to measure physician competence in shared decision making based on self-assessment. However, in my opinion, they should put some practical or real-world application based on their newly claimed method especially in abstract, discussion, and conclusion. This article was only provide many theoretically methods without their application in the real-world setting.

Thank you very much for this suggestion. We have added a substantial amount of information on implications for practical and real-world applications to the revised manuscript (Abstract, Discussion, Conclusions).

#2 The language used in the article perhaps will be difficult to be followed by most readers since it is full of technical words without explanation the function and meaning. I suggest they can revise the language used.

Thank you for keeping the readers' technical skills in mind. We have added non-technical explanations to several points and revised the language substantially throughout the manuscript to improve comprehensibility.
