# Supplementary material for: Application of the skills network approach to measure physician competence in shared decision making based on self-assessment
Source: PLoS One. 2023 Feb 27;18(2):e0282283. doi: 10.1371/journal.pone.0282283 (PMC9970074; doi:10.1371/journal.pone.0282283)
Supplement: Attachment — Submitted filename: Response to reviewers.docx [file pone.0282283.s008.docx]

Response to reviewers

**EDITORIAL COMMENTS**

**E#1. Thank you for submitting your manuscript to PLOS ONE. After careful consideration, we feel that it has merit but does not fully meet PLOS ONE’s publication criteria as it currently stands. Therefore, we invite you to submit a revised version of the manuscript that addresses the points raised during the review process. I am aware that you have waited a long time for this decision, and I apologise for this. Unfortunately, the original Academic Editor and reviewers became unavailable, apart from reviewer 1, who has reassessed the manuscript and is very happy with the result, but asks only that all data is shared, as per PLOS ONE data sharing policies. As such, two new reviewers were invited, who only have minor suggestions to improve the strength of the manuscript.**

Response to E#1. Thank you for taking this quite long process in your hands. We will share the data as recommended by Reviewer 1 and have implemented the suggestions of the two new reviewers.

**E#2. Reviewer 5 provides detailed comments, which we ask that you consider carefully.**

Response to E#2. We have revised the manuscript according to the suggestions of Reviewer 5.

**E#3. Reviewer 4 suggests that the manuscript might be difficult for a layperson to utilise. This limitation would not preclude consideration for publication in PLOS ONE, and I leave this with you to assess whether you prefer to address this in the revised manuscript, or whether you choose to otherwise make the findings from your study accessible to a layperson audience or clinicians interested in the topic, such as through a preprint, on your own website, Figshare etc. You may also add this information as a "Comment" on the final paper at a later stage, should it be published in PLOS ONE.**

Response to E#4. We have substantially improved readability for laypersons in two peer review rounds and are very happy with the result of this process. However, a further tailoring of the contents for laypersons would endanger the accuracy of the technical language. Therefore, we have decided to create a plain language summary of the study and make it publicly accessible. We have included the link to this summary in the revised article.

**REVIEWER 1**

**R1#1. All comments have been addressed. Perhaps, complete data can be shared using data sharing platform such as figshare, etc.**

Response to R1#1. Thank you for reviewing the revised manuscript. We will share the data and the analysis code of the study.

**REVIEWER 4**

**R4#1. The authors indicate that they have added real world practical value, but it's not easy to glean. There remains a lot of technical and statistical jargon that would be difficult for a layperson to access.**

Response to R4#1. Thank you for reviewing the manuscript. We feel that a further tailoring of the contents for laypersons would endanger the accuracy of the technical language. Therefore, we have decided to create a plain language summary of the study and make it publicly accessible. We have included the link to this summary in the revised article.

**REVIEWER 5**

**R5#1. The paper has very interesting data and a well-organized analysis. The goal is to improve the SDM definition by understanding how physicians' behavioral skills could be organized into patterns to help patients make medical decisions.**

Response to R5#1. Thank you for this kind feedback and the helpful comments on the manuscript.

**R5#2. Measures: Their description of qualitative analysis is only briefly described. For instance, could you please elaborate on OPTION-5 and OPTION-12 measures? OPTION-5 is a shorter version of OPTION-12 what was the rationale to use both measures? What are the examples of the items?**

Response to R5#2. The study did not include qualitative analyses. Nevertheless, we have added information on the measures to the Methods section of the revised manuscript, including the rationale for using three measures.

**R5#3. Results: Could you please report the qualitative results of transcript coding using the standard guidelines (O’Brien et al., 2014)? What is interrater reliability? What were the key examples of observed competencies?**

**O’Brien, Bridget C. PhD; Harris, Ilene B. PhD; Beckman, Thomas J. MD; Reed, Darcy A. MD, MPH; Cook, David A. MD, MHPE. Standards for Reporting Qualitative Research: A Synthesis of Recommendations. Academic Medicine: September 2014 - Volume 89 - Issue 9 - p 1245-1251 doi: 10.1097/ACM.0000000000000388**

**Response to R5#3.** The study did not include qualitative analyses, but we have added findings on the interrater reliability of the measures.

**R5#4. SDM Q9 and Option 5; Option 12; Are created based on Ewyn’s model of SDM. How do the clusters identified in network analysis speak to this model?**

Response to R5#4. According to our knowledge, Elwyn and colleagues have not specified an explicit model of how certain skills interact, although they did group them into loosely defined categories (‘team talk”, “option talks”, “decision talk”). In our study, we did not attempt to identify skill clusters. We have included this interesting line of though in the Discussion of the revised manuscript.

**R5#5. How stable are discovered patterns in the network? If physician rating is regressed only on transcripts data of patients with diabetes, or only with depression, do you see the same patterns?**

Response to R5#5. This is a very important question. Unfortunately, the amount of data that were available for analysis is insufficient for performing subgroup analyses. Nevertheless, we added to the Discussion of the revised manuscript that possible moderators of the findings should be explored further.

**R5#6. Discussion: Network analysis shows that relationships between skills need to be considered to ensure that skills could predict observable competencies. To what extent do specific patterns matter in this analysis versus the presence or absence of skills? How could the discovery of patterns in the physicians’ skills contribute to physicians’ education and practice?**

Response to R5#6. If our results are replicated and prove to be robust in independent studies, this might be one of the most interesting implications of our findings. It would be possible to create a skills network for an individual physician or student (based on at least ten, better more, consultations) and pinpoint precisely the skill or the connection between skills that needs to be improved. This could lead to a data-driven individualized training and monitoring competences, showing the way towards a kind of “precision medical education”. We have included this thought in the Discussion of the revised manuscript.
